# The immune score as a new possible approach for the classification of cancer

**DOI:** 10.1186/1479-5876-10-1

**Published:** 2012-01-03

**Authors:** Jérôme Galon, Franck Pagès, Francesco M Marincola, Magdalena Thurin, Giorgio Trinchieri, Bernard A Fox, Thomas F Gajewski, Paolo A Ascierto

**Affiliations:** 1 INSERM, U872, Laboratory of Integrative Cancer Immunology, 15 Rue de l'Ecole de Medecine F-75006 Paris, France; 2 Université Paris Descartes, Paris, 45 Rue Saints Pères 75006 Paris, France; 3 Centre de Recherche des Cordeliers, Université Pierre et Marie Curie Paris 6, 15 rue de l'Ecole de medecine F-75006 Paris, France; 4 Assistance Publique-Hopitaux de Paris, HEGP, 20-40 rue Leblanc, 75015 Paris, France; 5 Society for ImmunoTherapy of Cancer, Milwaukee, 555 East Wells Street, Suite 1100 | Milwaukee, WI 53202-3823 USA; 6 Infectious Disease and Immunogenetics Section (IDIS), Clinical Center and trans-NIH Center for Human Immunology (CHI), National Institutes of Health, Bethesda, Maryland, USA 20892; 7 Cancer Diagnosis Program, National Cancer Institute (NCI), National Institutes of Health (NIH), Rockville, Maryland, 20852, USA; 8 Cancer and Inflammation Program, Laboratory of Experimental Immunology, Center for Cancer Research, National Cancer Institute, National Institutes of Health, Bldg. 560/Room 31-93, Frederick, Maryland 21702, USA; 9 Laboratory of Molecular and Tumor Immunology, Earle A. Chiles Research Institute, Robert W. Franz Cancer Center, Providence Portland Medical Center, Portland, OR 97213, USA; 10 Department of Molecular Microbiology and Immunology, Oregon Health and Science University, 3181 SW Sam Jackson Park Road L220 Portland, OR 97239, USA; 11 Department of Pathology and Department of Medicine, Section of Hematology/Oncology, the University of Chicago, Chicago, IL 97239, USA; 12 Medical Oncology and Innovative Therapies Unit, Istituto Nazionale per lo Studio e la Cura dei Tumori, Fondazione "G. Pascale", Via Cappella dei Cangiani 1, 80131Napoli, Italy; 13 Fondazione Melanoma Onlus Via Mariano Semmola, 80131 Napoli, Italy

## Abstract

The outcome prediction in cancer is usually achieved by evaluating tissue samples obtained during surgical removal of the primary tumor focusing on their histopathological characteristics. Tumor staging (AJCC/UICC-TNM classification) summarizes data on tumor burden (T), presence of cancer cells in draining and regional lymph nodes (N), and evidence for metastases (M). However, this classification provides limited prognostic information in estimating the outcome in cancer and does not predict response to therapy. It is recognized that cancer outcomes can vary significantly among patients within the same stage. Recently, many reports suggest that cancer development is controlled by the host's immune system underlying the importance of including immunological biomarkers for the prediction of prognosis and response to therapy. Data collected from large cohorts of human cancers demonstrated that the immune-classification has a prognostic value that may be superior to the AJCC/UICC TNM-classification. Thus, it is imperative to begin incorporating immune scoring as a prognostic factor and to introduce this parameter as a marker to classify cancers, as part of the routine diagnostic and prognostic assessment of tumors. At the same time, the inherent complexity of quantitative immunohistochemistry, in conjunction with variable assay protocols across laboratories, the different immune cell types analyzed, different region selection criteria, and variable ways to quantify immune infiltration underscore the urgent need to reach assay harmonization. In an effort to promote the immunoscore in routine clinical settings worldwide, the Society for Immunotherapy of Cancer (SITC), the European Academy of Tumor Immunology, the Cancer and Inflammation Program, the National Cancer Institute, National Institutes of Health, USA and "La Fondazione Melanoma" will jointly initiate a task force on Immunoscoring as a New Possible Approach for the Classification of Cancer that will take place in Naples, Italy, February 13^th^, 2012. The expected outcome will include a concept manuscript that will be distributed to all interested participants for their contribution before publication outlining the goal and strategy to achieve this effort; a preliminary summary to be presented during the "Workshop on Tumor Microenvironment" prior to the SITC annual meeting on October 24^th ^- 25^th ^2012 in Bethesda, Maryland, USA and finally a "Workshop on Immune Scoring" to be held in Naples in December of 2012 leading to the preparation of a summary document providing recommendations for the harmonization and implementation of the Immune Score as a new component for the classification of cancer.

## Introduction

Conventional clinical and pathological risk prediction in cancer patients is usually achieved by evaluating tissue samples obtained during surgical removal of the primary tumor, mostly focusing on their histopathological characteristics. These can include the size of the tumor within the tissue, atypical cell morphology, tissue integrity, aberrant expression of protein and recently genetic markers or other evidence of malignant transformation, senescence and proliferation, various characteristics of the invasive margin, depth of invasion, and the extent of vascularization. In addition, histological or radiological analysis of both, tumor draining- and regional lymph nodes, as well as of distant organs are carried out looking to identify evidence of metastases. In accordance with these staging data, the evaluation of cancer progression is performed longitudinally and is further applied to estimate the patient prognosis. Available statistical data of patients with similar progression characteristics and their actual outcome parameters such as average disease-free (DFS), disease-specific (DSS) and overall survival (OS) are used for the estimation. Tumor staging (AJCC/UICC-TNM classification) summarizes data on tumor burden (T), presence of cancer cells in draining and regional lymph nodes (N) and evidence for metastases (M). Until now, this classification which is only based on tumor invasion parameters has been shown to be valuable in estimating the outcome of patients with a variety of cancers [1-3].

Still, it is well known that it provides limited information for prognosis since cancer outcome can significantly vary among patients within the same histological tumor stage. In some patients, advanced stage cancer can remain stable for years, and partial or full regression of metastatic tumors can occur spontaneously [4], although this is rare. On the other hand, rapid progression and death of patients with early cancers is often seen, even after complete surgical removal of the macroscopically visible tumor, and with no evidence of residual tumor burden or distant metastasis [4].

One reason for the apparently limited predictive accuracy of the traditional staging system is the assumption that tumor progression is largely a cell-autonomous process, focusing only on cancer cells and without properly considering the host immune response [5]. However, histopathological analysis has revealed that tumors are often infiltrated by a variable degree of inflammatory and lymphocytic cells [6]. A closer look reveals that these immune cells are not distributed randomly, but seem to be organized in more or less dense infiltrates in the center of the tumoral zone (CT), at the invasive margin (IM) of tumoral nests and in lymphoid islets adjacent to the tumor. The presence of immune cells may reflect a distinct underlying biology of the tumor, as gene expression profiling and confirmatory assays have revealed the presence of a broad signature of inflammation. This signature includes evidence for innate immune activation, chemokines for T cell recruitment, immune effector molecules, and expression of immune regulatory factors [7-9].

### A new classification of cancer based on the tumor microenvironment

Recently, multiple articles [8,10-13] and meeting reports [14-16] were published supporting the hypothesis that cancer development is influenced by the host immune system. This underlines the importance of including systemic and local immunological biomarkers that should be evaluated even in clinically observable tumors for making decisions regarding patients' prognosis and prediction to treatment [5,17]. Numerous data collected from large cohorts of human cancers demonstrated that the number, type and location of tumor immune infiltrates in primary tumors are essential prognostic factor for disease-free and overall survival. Global analysis of the tumor microenvironment showed that the immune contexture [10], i.e. the nature, functional orientation, density, and location of adaptive immune cells within distinct tumor regions influence the risk of relapse. An immune-classification of tumors was proposed based on a simple immune score, quantifying the density and location of immune-cells within the tumor [11]. This immune-classification has a prognostic value that may be superior to the AJCC/UICC TNM-classification, and tumor invasion was shown to be, in fact, statistically dependent on the host-immune reaction. Indeed, the immune pattern remained the only significant criterion over the classical AJCC/UICC TNM classification for disease free and overall survival, and led to an editorial entitled "TNM staging in colorectal cancer: T is for T cell and M is for memory" accompanying the publication by Mlecnik et al. in the Journal of Clinical Oncology [11,18].

These results suggest that once human cancer becomes clinically detectable, the adaptive immune response plays a role in preventing tumor recurrence. The ability of effector-memory T cells to recall previously encountered antigens leads to a protective response. Following a primary exposure to antigen, memory T cells disseminate and are maintained for long periods [19]. The trafficking properties and the long-lasting antitumor capacity of memory T cells could result in long-term immunity in human cancer.

Although first described in colorectal cancer, the impact of the immune cytotoxic and memory T cell phenotype has been demonstrated in many other human tumors and appears to be a general phenomenon [17,20]. It is interesting to note that the value of this immune phenotype applies not only to various organs of cancer origin (breast, colon, lung, head and neck, kidney, bladder, ovary, prostate...), but also to various cancer cell types (adenocarcinoma, squamous cell carcinoma, large cell cancer, melanoma, etc).

### The Immune Score as a new approach for the classification of cancer

Considering the probable universal character of the immune control of tumors, it is essential to begin to incorporate the immune score as a prognostic factor [17] and a component of cancer classification [11,13]. This marker has a dual advantage: firstly, it appears to be the strongest prognostic factor for disease free and overall survival particularly in early stage cancers, and secondly, it provides a tool or a target for novel therapeutic approaches, including immunotherapy. Current immunohistochemical technologies allow the application of such analyses by laboratories concerned with routine diagnostic and prognostic assessment of tumors.

The inherent complexity of immunohistochemistry, in conjunction with variable assay protocols across laboratories, the different immune cell types analyzed, different region selection criteria, qualitative and semi-quantitative criteria to measure immune infiltration contribute to the variability of the results obtained, and raise the concern that specialized protocols and training may be required. Therefore, it is essential to pursue assay harmonization to reduce these limitations. Many markers, signatures, and methods have been described to evaluate the prognosis of cancer patients. Yet, very few such markers and laboratory assays are used in clinical practice. Thus, we believe that harmonization of a assays evaluating the "inflammation", i.e. the immune score of the patient is essential. Although there is a need for analytical and clinical validation of the assay before the immune score for individual patient will reach clinical applicability, the current immunohistochemical technologies allow the application and cross-validation of such analysis in laboratories performing routine diagnostic and prognostic assessment of tumors. We expect a consensus panel to agree on a standardized set of assays in order to be able to compare results in the future, and for developing more effective prognostic and predictive markers to improve clinical decision-making in the care of cancer patients. Additional markers could be added subsequently to refine even further the method. Assay harmonization should minimize data variability and allow worldwide correlations of immune score results with clinical outcomes. Harmonization guidelines resulting from this process are expected to be simple to implement, and will improve assay performance [21].

A very important clinical translation is the establishment of an immune score based on the density of two lymphocyte populations, cytotoxic and memory T cells (CD8/CD45RO), both in the center and the invasive margin of tumors, to establish prognosis of clinical outcome in patients, even when there is no cancer associated prognostic marker such as in early tumor stage (I/II) patients [13]. In human cancers, a high density of T_H_1/cytotoxic memory T lymphocytes located both in the center and the invasive margin of the primary tumor is associated with long disease free and overall survival and low risk of relapse and metastasis. This was particularly illustrated in colorectal cancer [4,8,11-13], and should be applicable to most human tumors [17]. Thus, this immune score classification may help identify the high-risk patients who would benefit the most from adjuvant therapy.

### An open access call for a broad participation to the development of a task force dedicated to the evaluation of the role of Immune Scoring of cancer

Over the past few years, the area of immune regulation at the level of the tumor microenvironment has gained a forefront position in cancer research, in melanoma [22] and all other cancer types. At the same time, advances have been made in the development of the immunoscore as a prognostic factor. In an effort to promote the utilization of such immunoscore in routine clinical settings worldwide, the Society for Immunotherapy of Cancer (SITC), the European Academy of Tumor Immunology (EATI), the Cancer and Inflammation Program, the National Cancer Institute, National Institutes of Health, USA and "La Fondazione Melanoma Onlus" will initiate a task force on "Immunoscoring as a New Possible Approach for the Classification of Cancer" that will take place in Naples, Italy, February 13th, 2012. The working group is aimed at identifying a strategy for the organization of worldwide voluntary participation by various groups and societies including but not limited to members of the World Immunotherapy Council that currently include the Biotherapy Development Association (BDA), Canadian Cancer Immunotherapy Consortium (CCIC), Cancer Immunotherapy Consortium (CIC) of the Cancer Research Institute (CRI), Cancer Immunotherapy Society (CIMT), the Chinese Society for Clinical Oncology (CSCO), Committee for Tumor Immunology and Bio-therapy (TIBT), Dutch Tumor Immunology Working Party (DTIWP), European Academy of Tumor Immunology (EATI), European Society for Cancer Immunology and Immunotherapy (ESCII), Italian Network for Tumor Biotherapy (NIBIT), Japanese Association of Cancer Immunology (JACI), Nordic Center for Development of Anti-tumour Vaccines (NCV-network), Progress in Vaccination Against Cancer (PIVAC), and the Tumor Vaccine and Cell Therapy Working Group (TVACT). These groups share a clinical or basic interest in the immune biology of the cancer microenvironment and will collaborate to define guidelines to assess the validity of this new approach. Members of the World Immunotherapy Council will meet in February 2012 (subsequently to the task force meeting in Naples), to discuss participation in this process and will likely contribute to the discussion by arranging sessions at their annual meetings through 2012. Additionally, pathologist associations and other medical specialty groups will be invited to participate and/or are welcome to contact the members of the task force to be included. This editorial, in part, represents an effort to advertise this project, in an open access forum to obtain the broadest participation by all interested parties.

Because colorectal cancer has been most comprehensively studied and the prognostic significance of immunologic parameters has been best validated, special emphasis will be placed in this disease at the beginning. Other cancer types, including melanoma, will be discussed as well. An independent international consensus panel of laboratories with expertise in the field immune cell infiltrate evaluation in tumors is expected to discuss and agree on a cross-laboratory assay validation for the development of an immunoscore prognostic method.

The expected outcome of this effort will be: 1) a concept manuscript that will seek broad participation of potentially interested societies; 2) enhancing the topic of immune infiltrates as markers for tumor prognosis and/or response to therapy; 3) stimulate international collaborations between research and clinical investigators focusing on validation studies. The concept manuscript will seek broad participation of potentially interested societies. In as much as not all interested parties might be able to participate in person, an open access manuscript will facilitate involvement in subsequent efforts of additional experts who will have missed the first round.

In addition, a two-day workshop on the tumor microenvironment that will be held October 24-25, 2012 in Bethesda, MD in conjunction with the Annual Meeting of the Society for the Immunotherapy of Cancer (SITC)

The most important outcome of this activity will be the development of preliminary guidelines for the collaboration between organizations and societies dedicated to cancer immunotherapy, and/or other aspects of cancer biology relevant to its progression and treatment. In association with and preceding the Annual Meeting of "la Fondazione Melanoma Onlus", the "First Meeting on the Immunoscoring as a New Possible Approach in the Classification of Cancer" will be organized and will be held in Naples, December 2012.

## References

[B1] Locker GY Hamilton S Harris J Jessup JM Kemeny N Macdonald JS Somerfield MR Hayes DF Bast RC Jr ASCO 2006 update of recommendations for the use of tumor markers in gastrointestinal cancer J Clin Oncol 2006 24 5313 5327 10.1200/JCO.2006.08.2644 17060676

[B2] Sobin L Wittekind C TNM classification of malignant tumors 2002 New York: Wiley-Liss

[B3] Weitz J Koch M Debus J Hohler T Galle PR Buchler MW Colorectal cancer Lancet 2005 365 153 165 10.1016/S0140-6736(05)17706-X 15639298

[B4] Mlecnik B Bindea G Pages F Galon J Tumor immunosurveillance in human cancers Cancer Metastasis Rev 2011 30 5 12 10.1007/s10555-011-9270-7 21249426 PMC3044219

[B5] Bindea G Mlecnik B Fridman WH Pages F Galon J Natural immunity to cancer in humans Curr Opin Immunol 2010 22 215 222 10.1016/j.coi.2010.02.006 20207124

[B6] Finn OJ Cancer immunology N Engl J Med 2008 358 2704 2715 10.1056/NEJMra072739 18565863

[B7] Gajewski TF Louahed J Brichard VG Gene signature in melanoma associated with clinical activity: a potential clue to unlock cancer immunotherapy Cancer J 2010 16 399 403 10.1097/PPO.0b013e3181eacbd8 20693853

[B8] Galon J Costes A Sanchez-Cabo F Kirilovsky A Mlecnik B Lagorce-Pages C Tosolini M Camus M Berger A Wind P Type, density, and location of immune cells within human colorectal tumors predict clinical outcome Science 2006 313 1960 1964 10.1126/science.1129139 17008531

[B9] Wang E Miller LD Ohnmacht GA Mocellin S Perez-Diez A Petersen D Zhao Y Simon R Powell JI Asaki E Prospective molecular profiling of melanoma metastases suggests classifiers of immune responsiveness Cancer Res 2002 62 3581 3586 12097256 PMC2241738

[B10] Galon J Fridman WH Pages F The adaptive immunologic microenvironment in colorectal cancer: a novel perspective Cancer Res 2007 67 1883 1886 10.1158/0008-5472.CAN-06-4806 17332313

[B11] Mlecnik B Tosolini M Kirilovsky A Berger A Bindea G Meatchi T Bruneval P Trajanoski Z Fridman WH Pages F Galon J Histopathologic-based prognostic factors of colorectal cancers are associated with the state of the local immune reaction J Clin Oncol 2011 29 610 618 10.1200/JCO.2010.30.5425 21245428

[B12] Pages F Berger A Camus M Sanchez-Cabo F Costes A Molidor R Mlecnik B Kirilovsky A Nilsson M Damotte D Effector memory T cells, early metastasis, and survival in colorectal cancer N Engl J Med 2005 353 2654 2666 10.1056/NEJMoa051424 16371631

[B13] Pages F Kirilovsky A Mlecnik B Asslaber M Tosolini M Bindea G Lagorce C Wind P Marliot F Bruneval P In situ cytotoxic and memory T cells predict outcome in patients with early-stage colorectal cancer J Clin Oncol 2009 27 5944 5951 10.1200/JCO.2008.19.6147 19858404

[B14] Butterfield LH Disis ML Fox BA Lee PP Khleif SN Thurin M Trinchieri G Wang E Wigginton J Chaussabel D A systematic approach to biomarker discovery; preamble to "the iSBTc-FDA taskforce on immunotherapy biomarkers" J Transl Med 2008 6 81 10.1186/1479-5876-6-81 19105846 PMC2630944

[B15] Butterfield LH Palucka AK Britten CM Dhodapkar MV Hakansson L Janetzki S Kawakami Y Kleen TO Lee PP Maccalli C Recommendations from the iSBTc-SITC/FDA/NCI Workshop on Immunotherapy Biomarkers Clin Cancer Res 2011 17 3064 3076 10.1158/1078-0432.CCR-10-2234 21558394 PMC3096674

[B16] Tahara H Sato M Thurin M Wang E Butterfield LH Disis ML Fox BA Lee PP Khleif SN Wigginton JM Emerging concepts in biomarker discovery; the US-Japan Workshop on Immunological Molecular Markers in Oncology J Transl Med 2009 7 45 10.1186/1479-5876-7-45 19534815 PMC2724494

[B17] Pages F Galon J Dieu-Nosjean MC Tartour E Sautes-Fridman C Fridman WH Immune infiltration in human tumors: a prognostic factor that should not be ignored Oncogene 2010 29 1093 1102 10.1038/onc.2009.416 19946335

[B18] Broussard EK Disis ML TNM staging in colorectal cancer: T is for T cell and M is for memory J Clin Oncol 2011 29 601 603 10.1200/JCO.2010.32.9078 21245434

[B19] Sallusto F Geginat J Lanzavecchia A Central memory and effector memory T cell subsets: function, generation, and maintenance Annu Rev Immunol 2004 22 745 763 10.1146/annurev.immunol.22.012703.104702 15032595

[B20] Ascierto ML De Giorgi V Liu Q Bedognetti D Spivey TL Murtas D Uccellini L Ayotte BD Stroncek DF Chouchane L An immunologic portrait of cancer J Transl Med 2011 9 146 10.1186/1479-5876-9-146 21875439 PMC3175185

[B21] Fox BA Schendel DJ Butterfield LH Aamdal S Allison JP Ascierto PA Atkins MB Bartunkova J Bergmann L Berinstein N Defining the Critical Hurdles in Cancer Immunotherapy J Transl Med 2011 9 214 10.1186/1479-5876-9-214 22168571 PMC3338100

[B22] Ascierto PA De Maio E Bertuzzi S Palmieri G Halaban R Hendrix M Kashani-sabet M Ferrone S Wang E Cochran A Future perspectives in melanoma research. Meeting report from the "Melanoma Research: a bridge Naples-USA. Naples, December 6th-7th 2010" J Transl Med 2011 9 32 10.1186/1479-5876-9-32 21439082 PMC3078100

